# Long Head of the Biceps Tendon Augmentation Enhanced With a Bioinductive Collagen Patch for Massive and Retracted Rotator Cuff Tears

**DOI:** 10.1016/j.eats.2025.103679

**Published:** 2025-06-27

**Authors:** Hubert Laprus, Grzegorz Słota, Bartłomiej Juszczak, Roman Brzoska

**Affiliations:** a St. Luke’s Hospital, Bielsko-Biala, Poland; b University Children’s Hospital, Cracow, Poland

## Abstract

Massive and retracted rotator cuff tears are challenging to treat, with high rates of retear. Current surgical techniques strive to restore full function by addressing biomechanical complexities of these injuries. Additional use of long head of the biceps tendon augmentation and bioinductive patch may lower the retear rate by promoting healing of the construct. This Technical Note describes technique of combined double-row rotator cuff repair, long head of the biceps tendon augmentation, and a bio-inductive collagen patch as well as consisting of first results.

Rotator cuff tears (RCTs) are among the most common pathologies affecting the aging population.^1^^,^^2^ Untreated, full-thickness tendon tears typically enlarge over time. Resultant massive and retracted RCTs are particularly challenging to manage because increased tear size correlates strongly with poorer outcomes and construct failure.^3^

In the recent years, many rotator cuff repair techniques have evolved to restore function and reduce pain. Double-row repair, although more technically demanding, yields superior outcomes and lower retear rates compared with single-row repair.^4^ Some studies favor long head of the biceps tendon augmentation (LHBT+) over partial repair, demonstrating a lower retear rate in massive or irreparable RCTs.^5^^,^^6^ However, LHBT+ primarily improves healing at tendon-bone interface and does not address the tendon-muscle junction.

Over the past decade, interest has grown in the bioinductive, resorbable collagen patch to promote host tissue regeneration and provide an appropriate healing environment. This patch has shown very promising results in patients with massive RCTs^7^ and in vivo studies.^8^ To create a more stable reconstruction for massive and retracted RCTs and to reduce risk of retears, the authors decided to combine a double-row rotator cuff repair with LHBT+, along with the implantation of the bioinductive collagen patch. The aim of this technique is to improve reconstructive stability and enhance the biological healing process.

## Surgical Technique

### Indications

Patients eligible for this have massive and retracted RCTs according to the definitions of Gerber et al.^9^ and Davidson and Burkhart^10^ and retracted to Patte degree 3.^11^ The technique is most commonly applied in patients with massive, post-traumatic RCTs characterized by significant tendon retraction in which the LHBT remains intact and without erosion. For those patients, considering the age and quality of the tendons, rotator cuff reconstruction is often a single-chance procedure; therefore, both augmentation techniques to reduce retear risk are valuable.

### Setup

**Fig 1:**
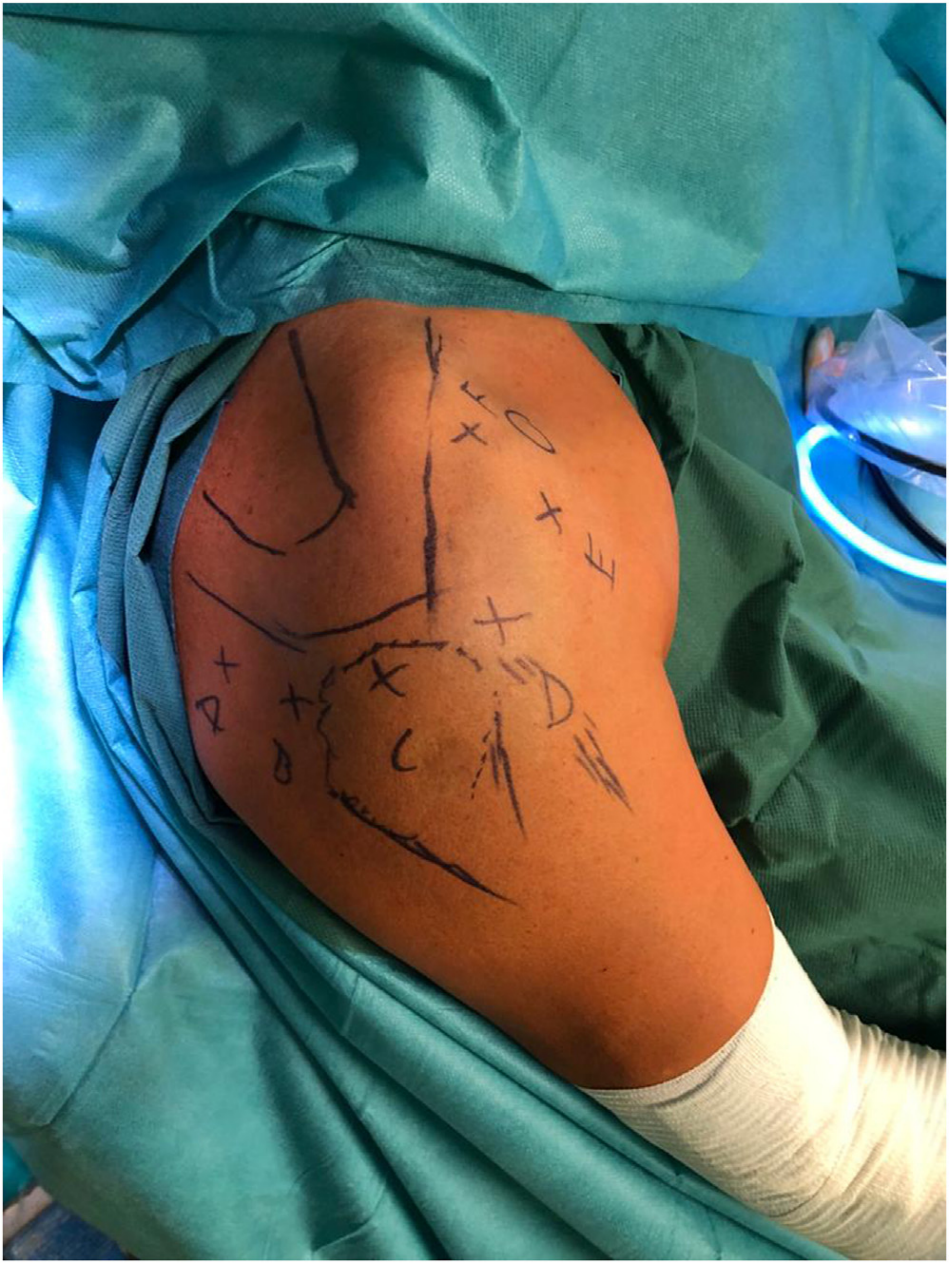
Arthroscopic portals used for the technique. The patient is positioned in a beach-chair position, right shoulder. (A) Posterior portal. (B) Posterolateral portal. (C) Lateral portal. (D) Anterolateral portal (over LHBT). (E) Anterior portal. (F) Additional anterosuperior portal. (LHBT, long head of the biceps tendon.)

The surgery is performed with the patient in the beach-chair position under general anesthesia in conjunction with an interscalene block. The patient’s head is aligned with the main axis of the body and securely stabilized with a head holder. The affected arm is positioned in a hand holder with traction to expand the subacromial space. An isotonic arthroscopic fluid solution containing 1 mL of adrenaline per 5 liters of fluid is infused to reduce potential bleeding. To avoid potentially damaging the functional posterior part of the rotator cuff tendon, a lateral, rather than the typical posterior, portal is used to set up the procedure (Fig 1). Complete bursectomy is performed, along with LHBT release in the bicipital groove and coracohumeral ligament release. Subsequently, rotator cuff tendons are debrided carefully with precise tendon layer differentiation and release both from the bursal and joint sides. The supraspinatus and infraspinatus tendons are completely mobilized by performing a 180° capsulotomy from the anterior to posterior glenoid margin, proceeding deep enough to visualize muscle fibers. Acromioplasty is only performed if full visualization is not achieved after bursectomy. The extent of tendon mobilization is assessed from the lateral portal by attempting to reposition the released structures to their anatomical landmarks.

### LHBT Augmentation

**Fig 2:**
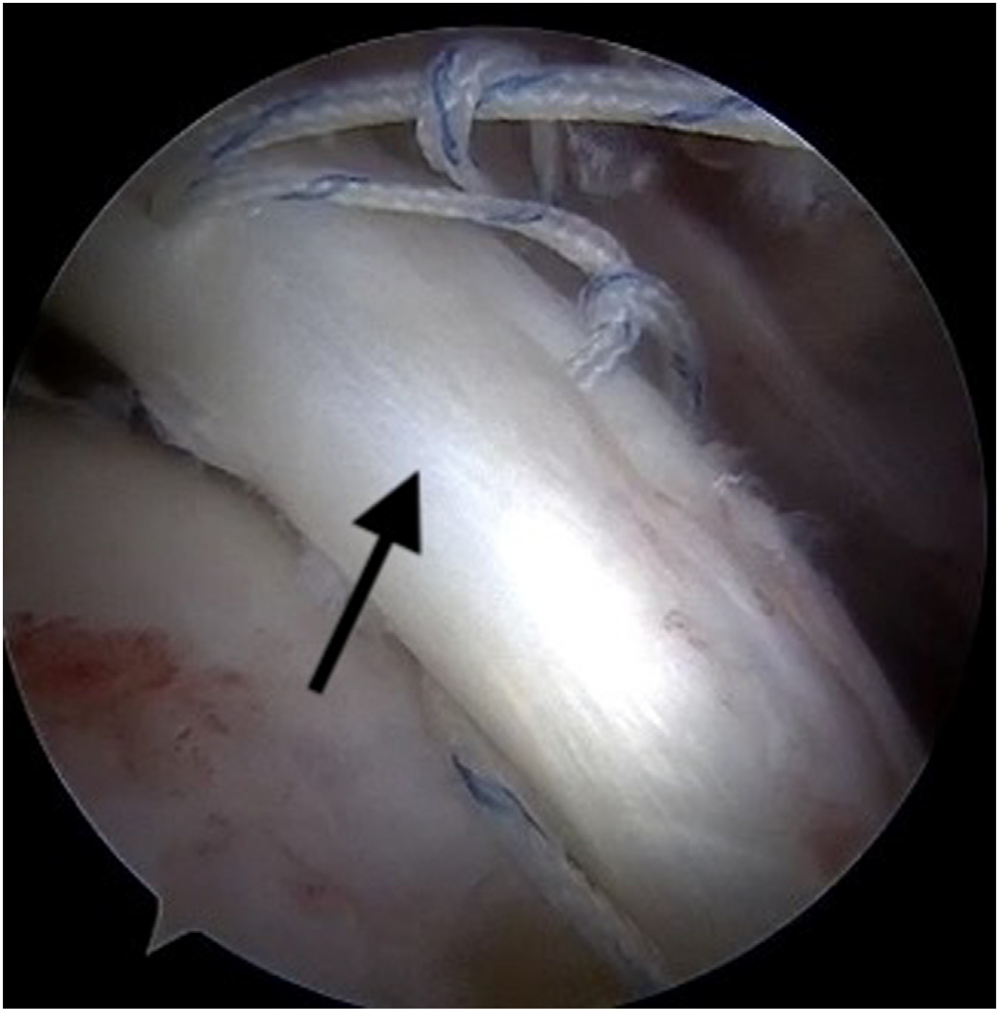
Long head of the biceps (black arrow) tenodesis in bicipital groove, made by a double-loaded anchor and hidden (double) lasso-loop sutures. View from the anterolateral portal.

**Fig 3:**
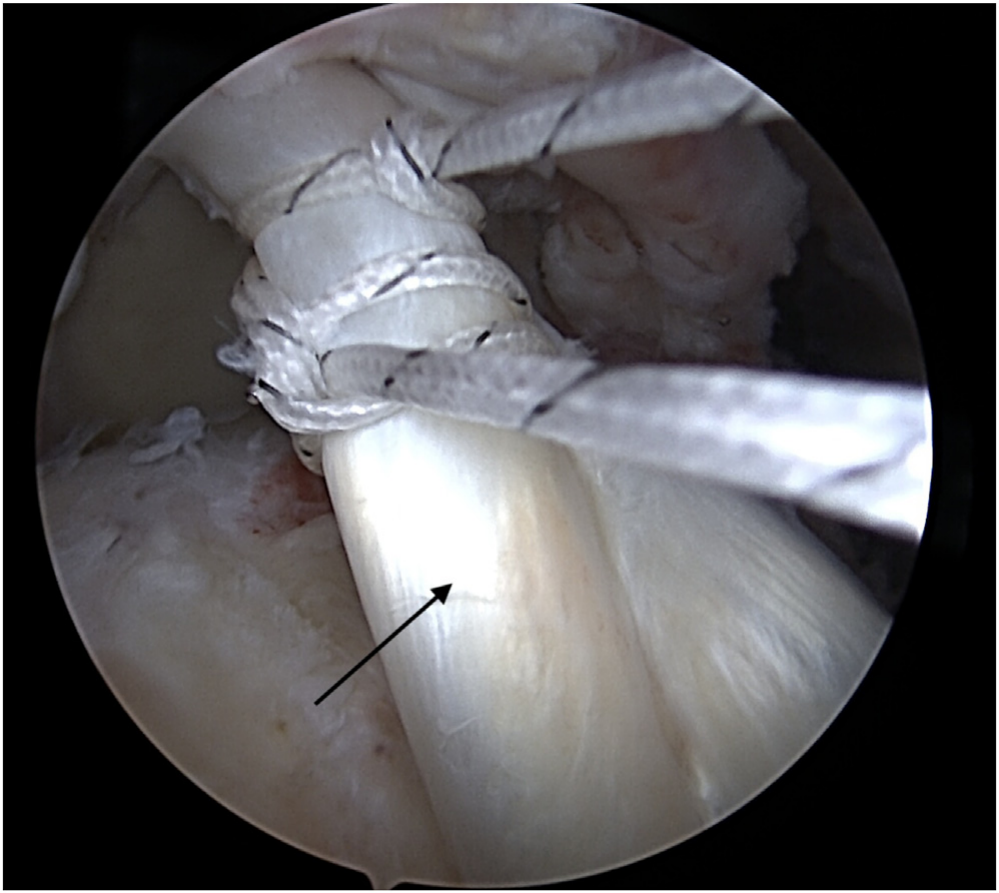
After tenodesis, the LHBT (black arrow) is encircled and sutured by 2 Krackow sutures using the thread from anchor used for tenodesis. View from the anterolateral portal. (LHBT, long head of the biceps tendon.)

**Fig 4:**
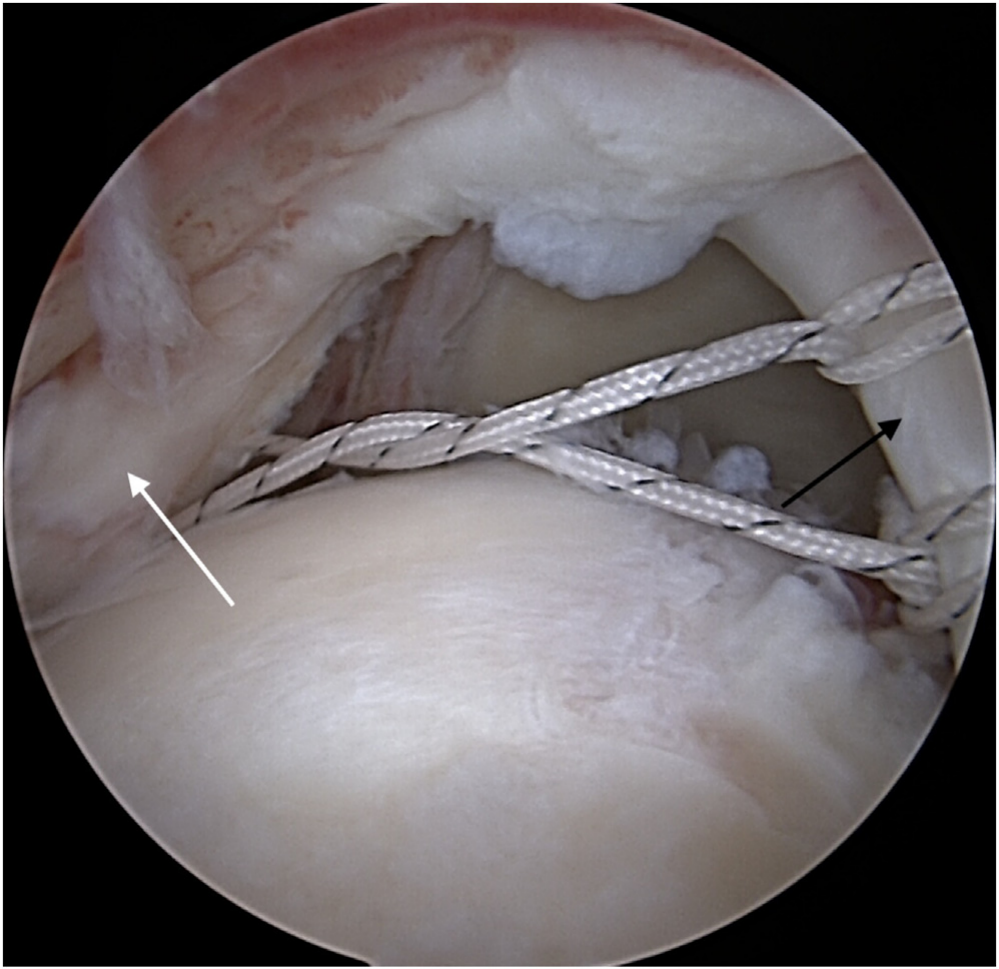
Threads with Krackow sutures from LHBT (black arrow) are passed through deep layer of infraspinatus tendon (white arrow) on the level, which will be approximate to reconstruct posterior rotator cable insertion. View from the anterolateral portal. (LHBT, long head of the biceps tendon.)

**Fig 5:**
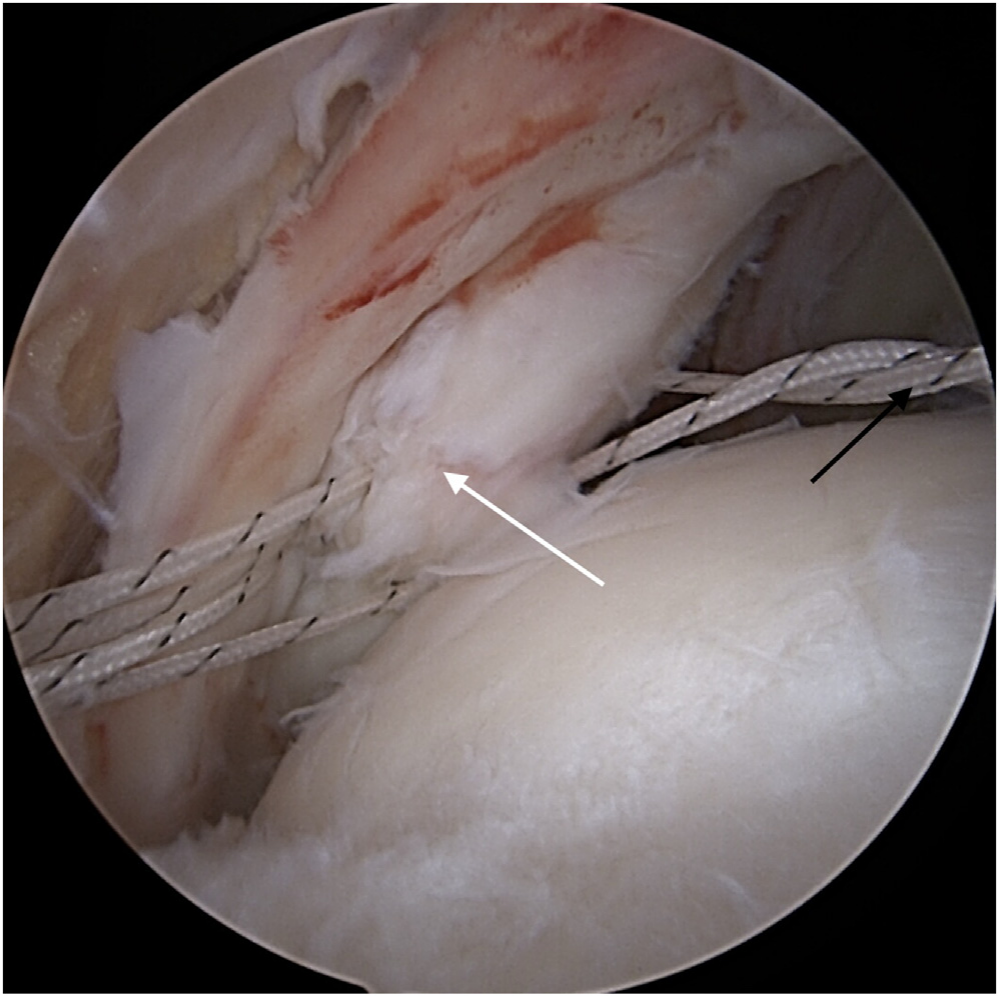
Second thread from the anchor used for LHBT tenodesis is passed through deep layer of the ISP (white arrow). Now, sutures from the LHBT (black arrow) and additional sutures passed through the deep layer of ISP are retrieved through the posterolateral portal (B). View from the lateral portal. (LHBT, long head of the biceps tendon.)

**Fig 6:**
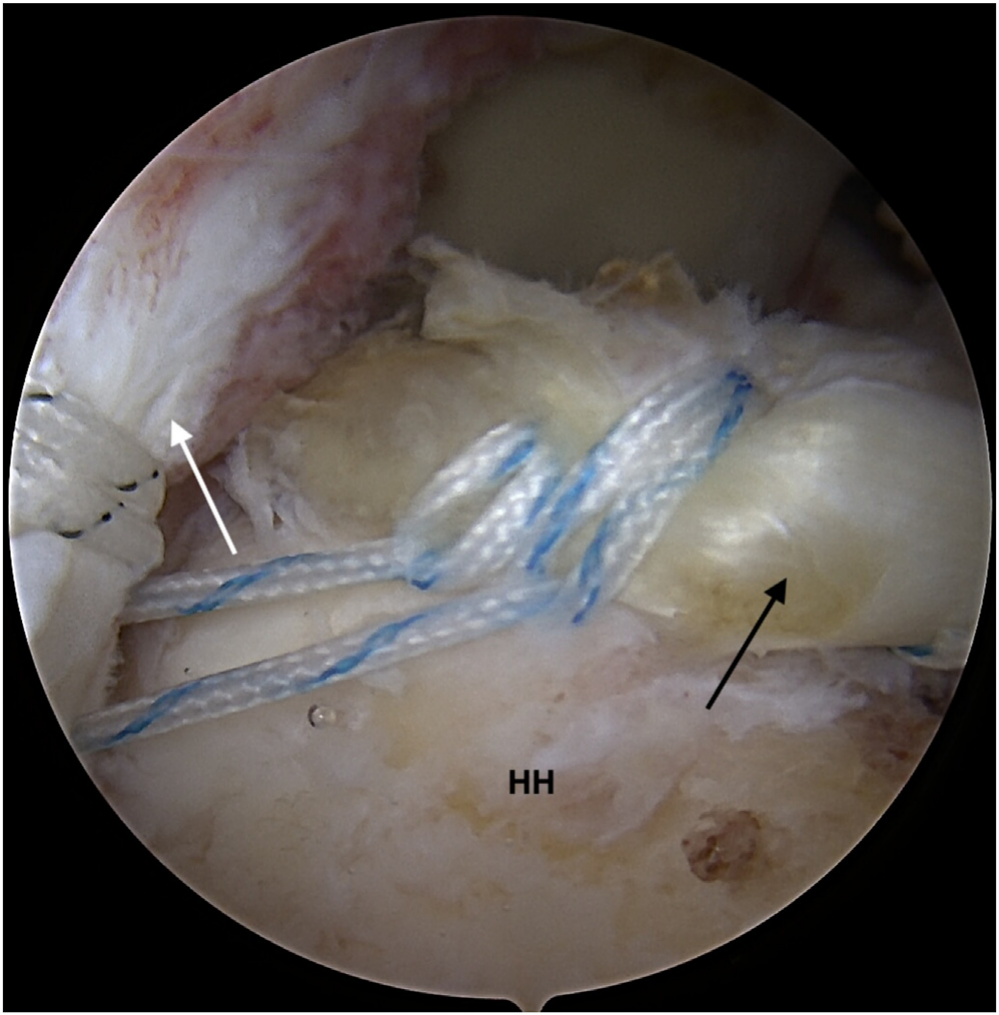
Biceps augmentation (black arrow) and deep layer of ISP (white arrow) are fixed by a knotless anchor. Both the biceps and the deep ISP layer are shown recreating a strong, “new” rotator cable construct at the edge of a cartilage of humeral head (HH). View from the lateral portal. Anchor fixation through posterolateral portal. (ISP, infraspinatus muscle tendon.)

Visualization of bicipital groove and LHBT assessment is achieved through the lateral and anteromedial working portals. Next, the greater tuberosity (and lesser in case of subscapularis muscle tear) and bicipital groove bone bed is prepared with a curette or high-speed burr to induce bleeding. Care should be taken to avoid excessive removal of greater tuberosity cortex to prevent pull-out of the implants. To reduce tension, a partial incision of the proximal attachment of LHBT is made. An all-suture double-loaded anchor is placed in bicipital groove and biceps tenodesis is performed using 2 lasso-loop sutures (Fig 2). In the case of subscapularis muscle tendon tear, another all-suture or titanium anchor is implanted in lesser tuberosity and the subscapularis tendon tear is repaired by single sutures or lasso-loop configuration. After tenodesis, LHBT is encircled with the first of remaining threads and a few Krackow sutures (Fig 3). Then, sutures from the LHBT are passed through deeper layer of the infraspinatus muscle tendon (ISP) in place where we want the deep ISP layer to replicate the rear rotator cable insertion to greater tuberosity (Fig 4). The second remaining thread from LHBT tenodesis is also passed through deep layer of ISP using a lasso-loop suture (Fig 5). The proximal LHBT attachment is then fully released and repositioned toward the ISP attachment by tensioning the sutures. Both structures are then sutured to the native attachment of the deep ISP layer at the posterior aspect of the greater tuberosity near the cartilage-bone interface using a knotless anchor (Arthrex, Naples, FL). The knotless anchor facilitates optimal tension adjustment on both the biceps and the deep ISP layer, recreating a strong, “new” rotator cable construct (Fig 6). The free suture from the knotless anchor is used to provide additional reinforcement of the ISP tendon. This reconstructs the rotator cable, transforming a previously massive and potentially irreparable tear into a smaller, more manageable injury.

### Double-Row Repair and Patch Augmentation

**Fig 7:**
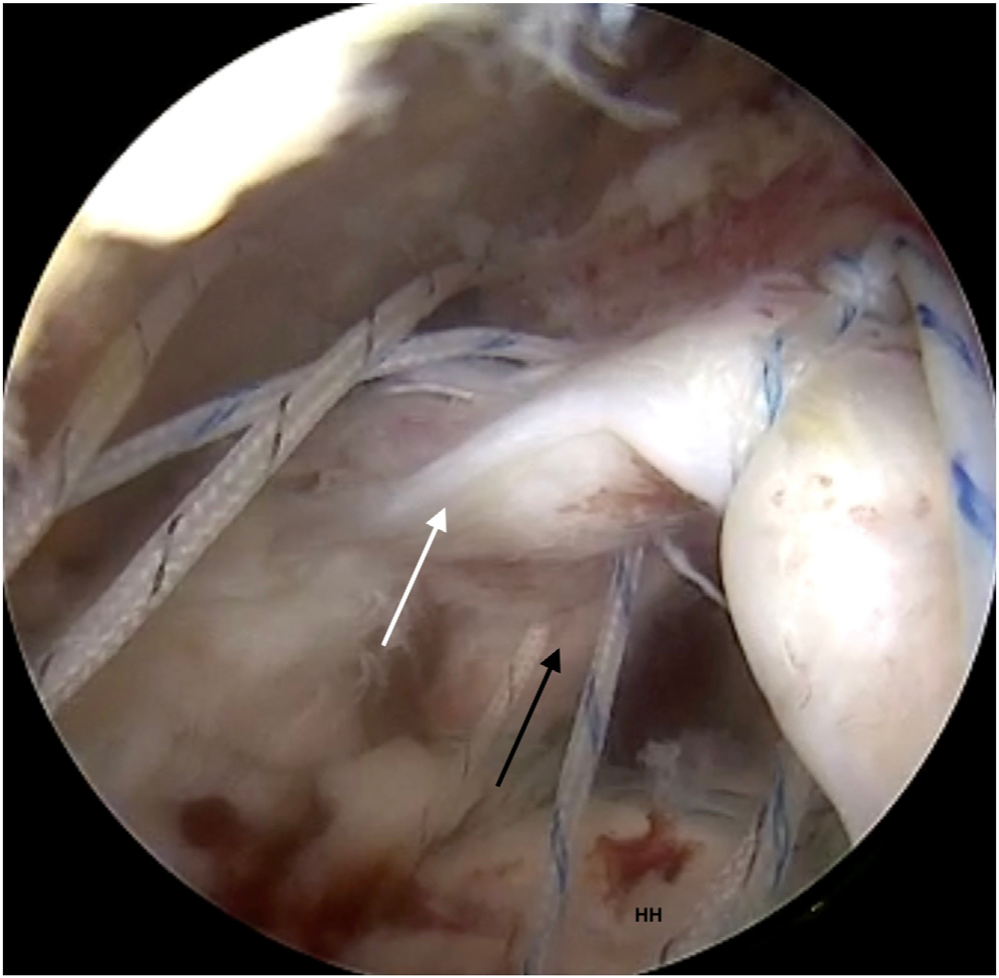
After LHBT augmentation and rotator cable reconstruction, double-layer reconstruction of the cuff is performed. It involves suturing the deep layer of the cuff (black arrow) using a lasso-loop suture, followed by passing the suture thread from the lasso-loop through the superficial layer (white arrow). The other end of the thread is passed through both tendon layers. View from lateral portal. (HH, humeral head; LHBT, long head of the biceps tendon.)

**Fig 8:**
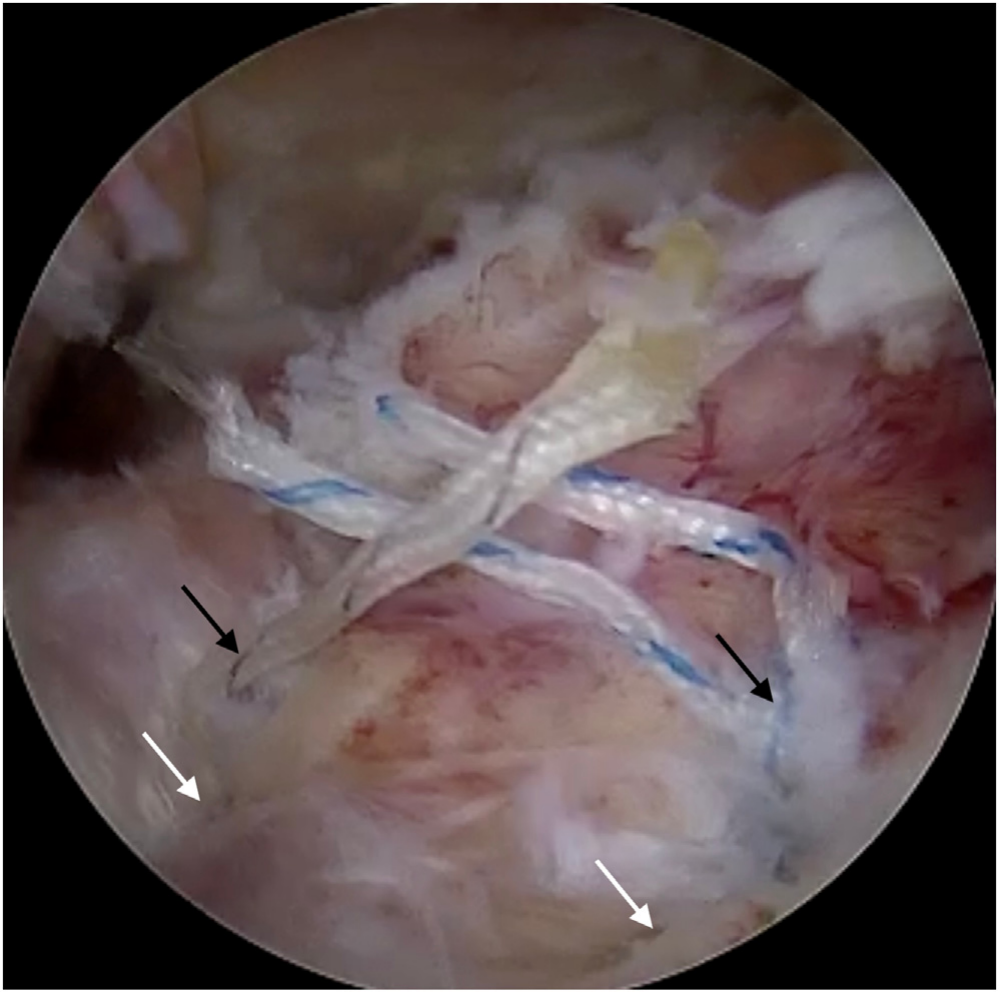
Final view of rotator cuff reconstruction after tying the knots. Black arrows indicates knots from first row, white arrows indicates sutures goes for second row anchor. View from the lateral portal.

**Fig 9:**
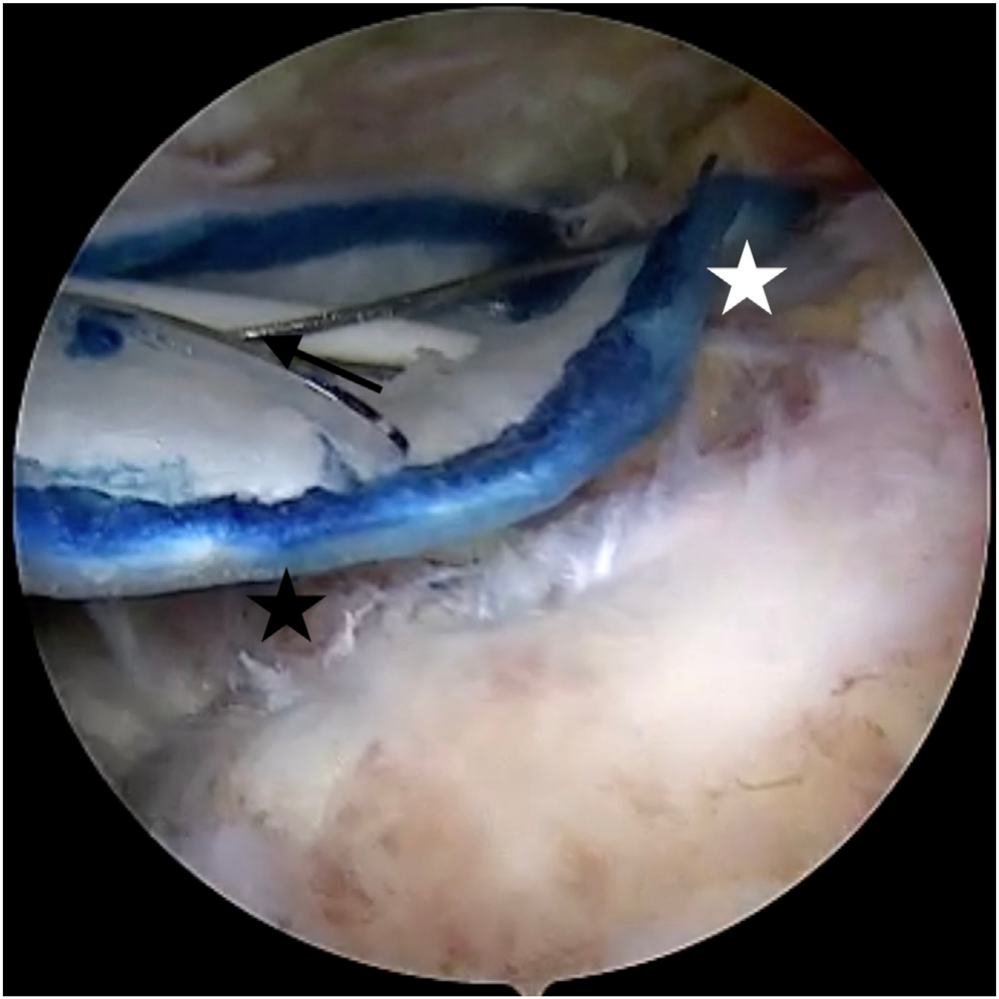
After rotator cuff reconstruction, bioinductive collagen patch (membrane with blue edges) is introduced through lateral portal by special expandable device (black arrow). Expandable device is used to press the patch against the tendon to facilitate subsequent fixation. Patch covers both the medial (musculotendinous junction: white star) and lateral (greater tuberosity footprint: black star) portions of the rotator. View from the anterolateral portal. Patch advanced through the lateral portal.

**Fig 10:**
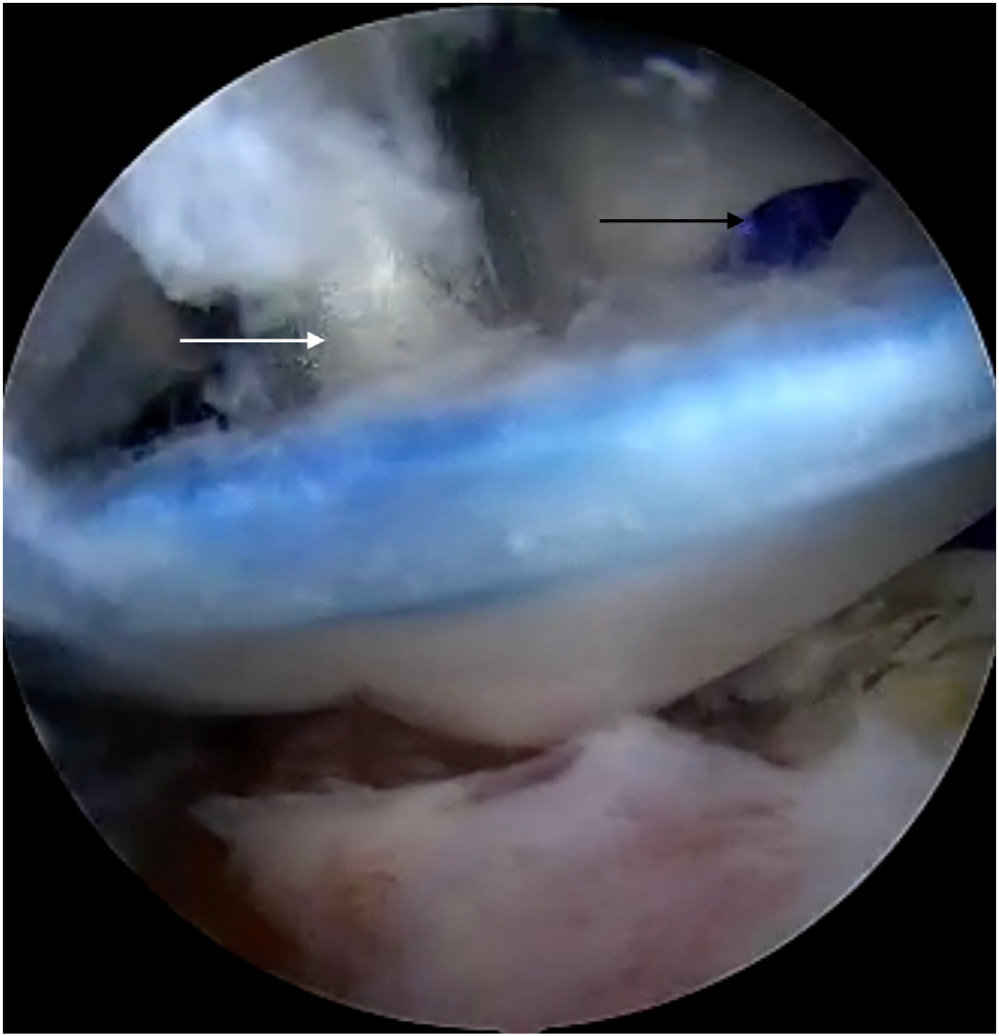
Patch is fixed to the tendon by absorbable blue stiches (black arrow), and then after removing expandable device, lateral (patch-bone) fixation is performed with the use of PEEK stiches and its inserter (white arrow). View from the anterolateral portal. Patch fixed to bone through lateral portal.

**Fig 11:**
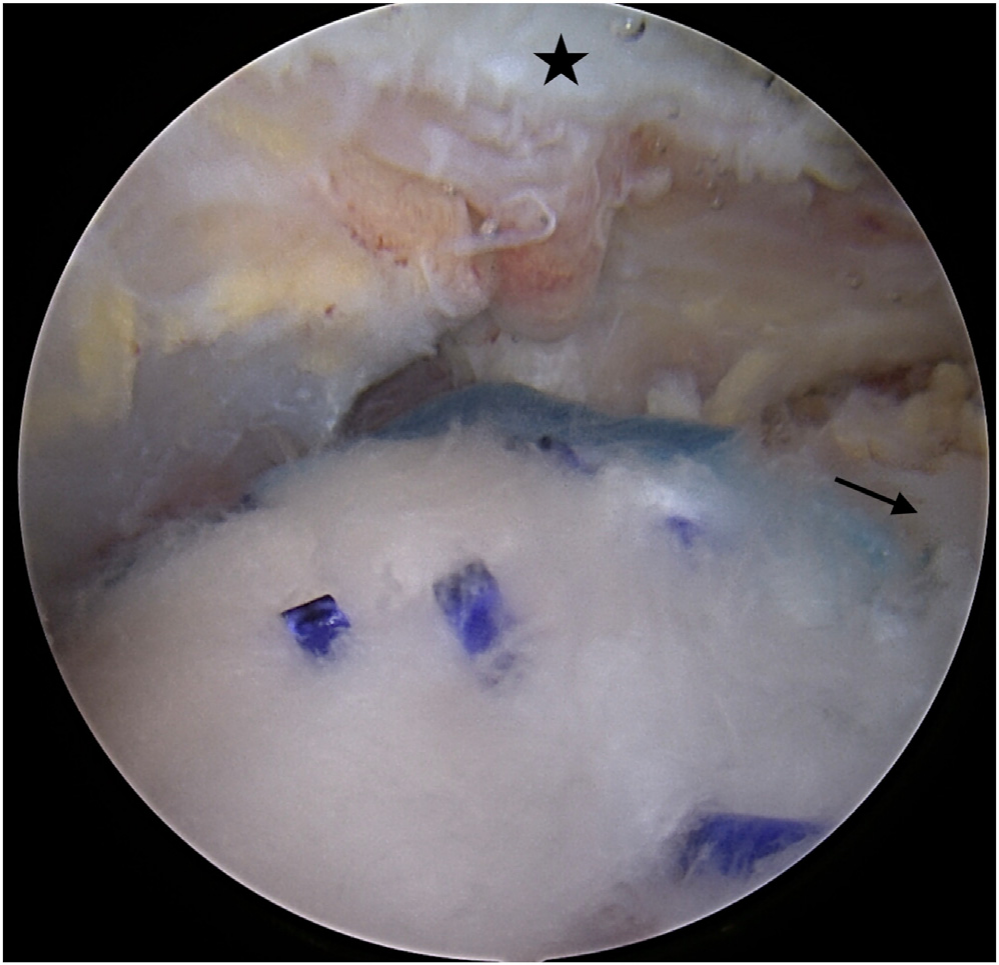
Final view after finishing total of procedure. Reconstructed rotator cuff (black arrow) is covered by collagen patch with blue absorbable stiches. Black star indicates acromion. View from the anterolateral portal.

After LHBT augmentation and reconstruction of posterior insertion of rotator cable, 2 additional titanium or all-suture anchors are used for a layered reconstruction of the supraspinatus and infraspinatus tendons. Layered reconstruction involves placing a lasso-loop suture through the deep layer of the cuff tendon and then repassing the same thread through the superficial layer. A second thread from the anchor is passed through both layers of the tendon (Video 1, Fig 7). After securing the first row of implants, further stabilization is achieved with knotless double-row anchors, covering the greater tuberosity with the reconstructed tendons (Fig 8). Next, to prepare for patch augmentation, a cannula used for absorbable stich placement from a set (Smith & Nephew, Memphis, TN) is inserted through the posterolateral portal. Then, a wider scaffold placement cannula (Smith & Nephew) is inserted through the lateral portal. Using the anterolateral portal for visualization, a specialized applicator with the scaffold (Smith & Nephew) is advanced over the reconstructed tendons through lateral portal with wider cannula. It is crucial to ensure the scaffold covers both the medial (musculotendinous junction) and lateral (greater tuberosity footprint) portions of the rotator cuff (Fig 9). First, bioabsorbable sutures (Smith & Nephew) secure the scaffold posteriorly and medially while simultaneously scaffold applicator pressing against the rotator cuff tendon and scaffold. The matrix is then stabilized and secured using a cannula or a magic grasper through the posterior portal, and the applicator is subsequently removed. A PEEK (polyether ether ketone) bone stapler applicator (Smith & Nephew) is introduced through the lateral portal, and the scaffold is anchored laterally to the bone of the greater tuberosity (Fig 10). Switching visualization to the lateral portal, the remaining bioabsorbable sutures are inserted through the anterolateral portal to achieve optimal scaffold tension and secure coverage of the reconstructed tendons (Fig 11, Video 1). Pearls and pitfalls of the technique as well as advantages and disadvantages are presented in Table 1 and Table 2, respectively.

**Table 1:** Pearls and Pitfalls of the Technique

Pearls	Pitfalls
Anterior to posterior capsulotomy up to level of muscle fibers is the key to achieving reconstruction without tension	Without capsulotomy, it’s difficult to impossible to reconstruct massive and chronic cuff with augmentation
Lateral bursectomy with abduction of the arm increases visualization and space needed to perform augmentation	If your thread for LHBT sutures for augmentation is too short, it will be hard to pass it through deep layer of ISP
During LHBT tenodesis, leave one thread from the anchor longer and use it later for suturing the LHBT for augmentation	If you pass sutures from augmentation too lateral through deep layer of ISP, you will cover footprint by LHBT (not enough place for cuff)
Before passing sutures through deep layer of ISP, pre-reduce the tendon to find proper place, usually is more medial than it seems to be	During fixation of augmentation, if you leave LHBT too loose, it can hinder proper cuff reconstruction
For augmentation use anchor with additional, strong thread, which will be used for ISP reconstruction	Be quick during patch implantation. After 10 minutes, it becomes soaked with water and it’s implantation is more difficult
During ISP-LHBT augmentation, stretch the LHBT as much as possible	
After reconstruction and before patch placement, check if you have enough space. If not perform acromioplasty.	

ISP, infraspinatus muscle tendon; LHBT, long head of the biceps tendon.

**Table 2:** Advantages and Disadvantages of the Technique

Advantages	Disadvantages
Greater stiffness and strength of the reconstruction thanks to the LHBT	More implants and additional patches generate greater costs of the procedure
Potentially protective effect of the LHBT on the reconstruction,	Longer time of surgery and increased difficulty
Improved healing potential attributable to collagen from the LHBT and the patch	

LHBT, long head of the biceps tendon.

## Discussion

This study describes a surgical technique for the treatment of massive and retracted RCTs, combining a double-row repair with (LHBT+) and the addition of a bioinductive collagen implant (REGENETEN; Smith & Nephew). The combined approach offers a comprehensive solution that addresses both mechanical stability and biological healing. The incorporation of LHBT augmentation appears to contribute to improved stability and vascularity of the construct, potentially reducing the risk of re-tear, a significant concern with massive rotator cuff repairs. Double-row repair together with LHBT+ helps reconstruct the rotator cable and ensure a strong and stable tendon footprint, which remains an important technical nuance of this procedure. The use of the bioinductive collagen implant aims to further enhance the healing process by stimulating tissue regeneration, especially at the musculotendinous junction where retears are the most devastating. This is supported by the positive tendon healing observed in most patients on postoperative magnetic resonance imaging. Nonetheless, the proposed technique has potential limitations. The need for an intact, well-quality LHBT may restrict its applicability. The technical complexity ad time required for the multilayered reconstruction with LHBT+ and scaffold application may limit widespread adoption as well as high cost of a matrix. This combined technique of LHBT augmentation, double-row repair, and bioinductive collagen patch application addresses both the mechanical and biological challenges posed by massive and retracted rotator cuff tears. Although technically demanding, it offers a promising treatment option for a particularly difficult patient population.

## Disclosures

All authors (H.L., G.S., B.J., R.B.) declare that they have no known competing financial interests or personal relationships that could have appeared to influence the work reported in this paper.

## Supplementary Data

Video 1 Surgical technique with commentary. The patient is positioned in the beach-chair position, right shoulder; the first viewing portal is lateral portal. Video consist of full debridement with technical pearls of tendon release, technique of LHBT tenodesis, LHBT augmentation with LHBT-ISP connection, rotator cable reconstruction, double-layer reconstruction of the rotator cuff and final augmentation with patch. Final viewing portal is the anterolateral portal. (ISP, infraspinatus muscle tendon; LHBT, long head of the biceps tendon.)
